# Public Discussion of Anthrax on Twitter: Using Machine Learning to Identify Relevant Topics and Events

**DOI:** 10.2196/27976

**Published:** 2021-06-18

**Authors:** Michele Miller, William Romine, Terry Oroszi

**Affiliations:** 1 Department of Pharmacology & Toxicology Wright State University Dayton, OH United States

**Keywords:** anthrax, big data, internet, infodemiology, infoveillance, social listening, digital health, biological weapon, terrorism, Federal Bureau of Investigation, machine learning, public health threat, Twitter

## Abstract

**Background:**

Social media allows researchers to study opinions and reactions to events in real time. One area needing more study is anthrax-related events. A computational framework that utilizes machine learning techniques was created to collect tweets discussing anthrax, further categorize them as relevant by the month of data collection, and detect discussions on anthrax-related events.

**Objective:**

The objective of this study was to detect discussions on anthrax-related events and to determine the relevance of the tweets and topics of discussion over 12 months of data collection.

**Methods:**

This is an infoveillance study, using tweets in English containing the keyword “Anthrax” and “*Bacillus anthracis*”, collected from September 25, 2017, through August 15, 2018. Machine learning techniques were used to determine what people were tweeting about anthrax. Data over time was plotted to determine whether an event was detected (a 3-fold spike in tweets). A machine learning classifier was created to categorize tweets by relevance to anthrax. Relevant tweets by month were examined using a topic modeling approach to determine the topics of discussion over time and how these events influence that discussion.

**Results:**

Over the 12 months of data collection, a total of 204,008 tweets were collected. Logistic regression analysis revealed the best performance for relevance (precision=0.81; recall=0.81; F_1_-score=0.80). In total, 26 topics were associated with anthrax-related events, tweets that were highly retweeted, natural outbreaks, and news stories.

**Conclusions:**

This study shows that tweets related to anthrax can be collected and analyzed over time to determine what people are discussing and to detect key anthrax-related events. Future studies are required to focus only on opinion tweets, use the methodology to study other terrorism events, or to monitor for terrorism threats.

## Introduction

### Background

Terrorism can be traced back to almost 2000 years ago when the Sicarii-Zealots, a Jewish resistance group, used assassins to stab Roman legionnaires or Jewish collaborators, use poison, or kidnap Temple Guard staff for ransom [1]. Terrorism can take many forms, and while each government agency has their own definition of terrorism, all agencies concur that the purpose is to instigate widespread fear in the target population [2-4].

The fear resulting from terrorism typically causes more damage to our economy and social fabric than the act of terror itself. Examples include an upsurge in hate crimes following the September 11, 2001, attacks on the World Trade Center (New York City, New York) and the fear of opening mailboxes after the anthrax attacks of 2001 [5,6]. Hence, in addition to neutralizing terror attacks before they occur, efforts to combat terrorism are also focused on minimizing negative social after-effects of attacks that do occur. To this end, it is imperative to identify and address fears and misconceptions to mitigate the additional damage.

Some terrorists utilize chemical, biological, radiological, and nuclear weapons or explosives (CBRNe) to instill fear. The anthrax attacks of 2001 are an example of bioterrorism where a biological weapon (anthrax) was used in the United States. The anthrax attacks are the only example of the use of a CBRNe agent in the United States. Nonetheless, there have been several anthrax hoaxes where people have received packages or envelopes with powder that is not anthrax. Considering the continued hoaxes and concern over an impending anthrax attack, it is important to continue to monitor for anthrax-related events.

Social media has facilitated studies on opinions and reactions to anthrax-related events in real time, thus eliminating the time lag and response bias associated with traditional survey methods. Infodemiology is the study of determinants and distribution of information on the internet, allowing data to be collected and analyzed in real time [7]. Infodemiology has enabled studies on public behavior and opinions during the COVID-19 pandemic [8,9], conspiracy theories [10], and public behavior and opinions during the Zika pandemic [11,12]. Anthrax, or *Bacillus anthracis*, is a gram-positive, rod-shaped, spore-forming, facultative anaerobic, aerobic, nonmotile bacterium [13]. Human anthrax infection occurs through three routes: gastrointestinal, cutaneous, and inhalation (pulmonary) [14]. Cutaneous anthrax is the most common but least dangerous form of infection, gastrointestinal anthrax has rarely been reported in the United States, and inhalation anthrax is considered the deadliest form. Irrespective of the route of infection, anthrax responds well to antibiotics when treated before the onset of symptoms [15-17]. Weapons-grade anthrax has been treated to reduce clumping, has a low electrostatic charge, a uniform particle size, and a high spore concentration [14]. An aerosol release of *B. anthracis* would be invisible and odorless, with the potential to travel several kilometers before dissipating [18]. This combination of a high infection rate, high virulence, and ease of spread makes anthrax an ideal bioweapon.

### Related Studies

Nordin et al [19], performed a computer simulation of uniform exposure to an anthrax release in the air intakes in the Mall of America (Bloomington, Minnesota). The completeness and timeliness of detecting the attack depended on the infection rate. The study by Nordin et al [19] improves upon detection using traditional methods and may allow natural outbreaks to be detected faster.

Mandl et al [20] suggested a 4-stage detection procedure for measuring outbreak detection using semisynthetic data sets. In the first stage, data were grouped by syndrome. In the modeling stage, historic data were used to understand temporal and spatial patient distributions. This was followed by the detection stage where predictions based on the model were compared to observed data. In the last stage, the health department determined if the outbreak was worth investigating on the basis of the large deviations observed in stage 3 [20].

The simulated anthrax epidemic injection model developed by Buckeridge et al [21] also consisted of four components: agent dispersion, infection, disease and behavior, and data sources. The models developed by Buckeridge et al [21] and Mandl et al [20] may also improve methods of detecting natural outbreaks and terrorism through their comparison with background noise. This study not only compared peak-to-background noise but also used real-time rather than historic data.

Kulldorf et al [22] used 3 different data sets to generate a null model, where each person in New York City is equally likely to contract anthrax, and 35 alternative models where 1 or more zip codes were assigned an increased risk on day 31, 32, or 33 post exposure. Kulldorf et al [22] reported that the statistical power was higher when more days had elapsed since the onset of the outbreak. Kulldorf et al [22] took a week to detect an event using simulated data.

The aforementioned studies are based on historic or simulated data and demonstrate how computers can improve event detection speed and precision compared to traditional survey methods. Furthermore, they explain how using naturally occurring events highlights the usefulness of their methods, but analysis using actual event data is needed. Real-time data encompassing 20 events that occurred will be used in this study. The aforementioned studies were also focused on detecting events from among physician visits, whereas event detection and the analysis of public opinions on the events were the focus of this study.

### Aims of the Study

**Figure 1:**
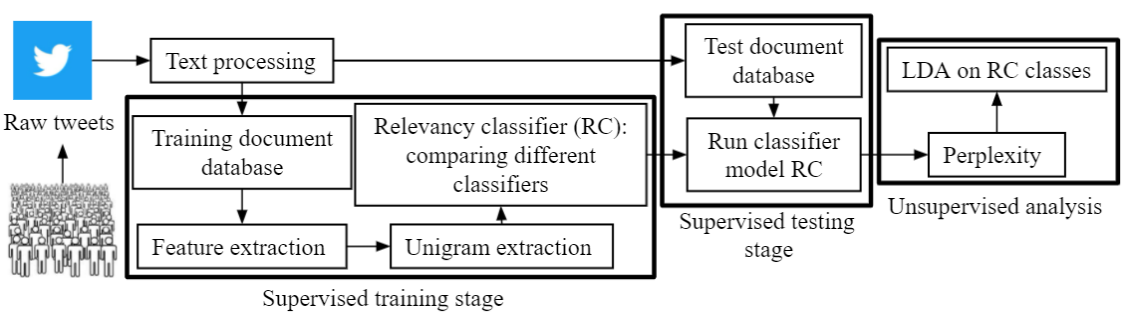
Methods for a hierarchical supervised classification technique. Large black boxes indicate where supervised machine learning algorithms were trained, supervised machine learning algorithms were tested, and where unsupervised machine learning algorithms were used. LDA: Latent Dirichlet allocation.

We aimed to carry out an exploratory analysis focused on developing a framework for detecting discussions on anthrax-related events on Twitter and topic modeling over several months. Using the methods shown in Figure 1, the following research questions were addressed: Event detection (R1): were discussions on anthrax-related events detected on Twitter? What events led to these discussions? Classification performance analysis (R2): what was the classification performance in detecting the tweets relevant to anthrax-related events? Topical analysis (R3): what were the main discussion topics during each month of data collection over a year-long period (September 25, 2017, to August 15, 2018)?

## Methods

### Methods Overview

A combination of natural language processing and machine learning techniques was used in this study to detect anthrax-related events and determine how tweets on anthrax change over time. Specifically, a classifier system was built for finding relevant tweets on anthrax and then categorizing them by month (Figure 1).

A primary concern of researchers using social media data is whether the data are public or private [23]. Tweets collected by using hashtags are generally considered public data since users are broadcasting their opinions to other users discussing the same topic [23]. For ethical reasons, tweets were not collected from specific accounts or through direct messages, the data were deindividualized, and usernames were removed from all collected tweets; therefore, all data collected were public data.

Tweets containing the keyword “Anthrax” and “*Bacillus anthracis*” in English were collected from September 25, 2017, through August 15, 2018, using the Twitter application programming interface (API) using real-time endpoints (Spyder Python 3.6). Tweets were collected in accordance with Twitter’s application programming interface documentation; hence, the tweets collected in this study constitute a subset as opposed to all tweets containing the keywords used. Data collected included text from 204,008 tweets as well as the date and time when the tweet was posted. These 204,0008 tweets included retweets and consisted of a random sample of all tweets containing 1 or both keywords.

### Event Detection (Addressing R1)

The number of tweets over time was plotted to detect anthrax-related events. If a 3-fold spike in tweets occurred within a 24-hour period, it was considered an event. A 3-fold spike was chosen because it allowed all spikes corresponding to anthrax-related topics to be detected but did not eliminate any important topics. Time of detection was determined as the time between an event occurring and when the first tweet regarding the event was detected. For all the events, the exact time of the event could not be determined; hence, the time when the first news article was posted or the time when a weblink reference in a tweet was posted was used instead. All times were converted to EST for delay calculations. Tweets about the Mueller investigation were a topic of discussion throughout data collection. At the time of the spikes, several tweets were highly retweeted along with other individual comments about the proceedings, which made it difficult to determine what caused the spikes in tweets. Therefore, the first highly retweeted tweet was used to determine the start of the spike and to obtain the article, weblink reference, or tweet that led to the spike.

### Classification Performance (Addressing R2)

A CBRNe expert and 2 data analysts trained by the expert annotated 5000 random unique tweets as “relevant” (scored as 1) or “not-relevant” (scored as 0) to create a gold standard data set. Cronbach α was used to evaluate interrater reliability between annotators using StataIC (version 15, Stata Corp).

If the tweet was about *B. anthracis*, it was considered relevant. For example, the tweet “RT: Remind me again, why did DC invade Iraq? Yellow cake and Nuclear weapons? Anthrax and Bio weapons? 9/11 Saudis?” was annotated as “relevant” since it mentions anthrax as one of the possible reasons why the United States invaded Iraq, whereas the tweet “Anthrax - In The End Official” was annotated as “not-relevant” because it refers to a song by the metal band “Anthrax.” The relevant tweets were then further categorized by the month and day when they were tweeted.

Before data analysis, the tweets had to be preprocessed by removing weblinks, hashtags, at-mentions, retweet indicators, and non-ASCII (American Standard Code for Information Interchange) characters. Data were further normalized by removing punctuation, numbers, uppercase letters, and white spaces. Terms such as single letters, stop words, and the search terms “anthrax” and “*Bacillus anthracis*,” which do not convey any additional meaning about the topics, were removed. Features included parts of speech (adjectives, singular nouns, past-tense verbs, past-participle verbs, verbs, determiners, prepositions, personal pronouns, plural nouns, singular proper nouns, predeterminers, and adverbs), the top 20 unigrams, and the top 20 bigrams. Feature codings were used to train the classifiers. All features were coded on the basis of the presence (scored as 1) or absence (scored as 0) of them in the tweet. The algorithms were then trained using the presence or absence codings for all features. All features were used for each machine learning algorithm.

Supervised machine learning algorithms including logistic regression, naïve Bayes classifier, support vector machine, and random forest were used for classifying relevance and events. These supervised methods rely on labeled data (tweets) to “learn” the nature of the tweets toward correctly classifying them. Tweets were categorized as relevant or not and then further divided by month.

The performance of each supervised algorithm was evaluated using 10-fold cross-validation, which serves to partition the data into 10 disjoint sets with equal samples from all classes [24]. The algorithm then trains on 10-1 of the sets and tests on the single hold-out set, repeating until all sets have been used for training 10-1 times and testing once. The performance of the machine learning algorithms was tested using a holdout of 500+1000 additional random tweets. Precision, recall, and F_1_-scores were calculated to test the performance on the holdout of 500+1000 additional tweets.

### Topical Analysis (Addressing R3)

Topic modeling using an unsupervised machine learning analysis was used to determine the most common topics of discussion during each month of data collection. Latent Dirichlet allocation (LDA) was chosen for topic modeling. LDA is an unsupervised machine learning technique that identifies the most common topics in tweets by clustering words with similar meanings [25]. In LDA, each document is represented by a mixture of topics, and each topic is represented by a mixture of words. In this study, topic modeling was used to determine the underlying topics for each month of data collection.

To determine the number of topics to include in the LDA analysis, the perplexity measure was chosen. Perplexity was used to evaluate the LDA results by testing the number of topic models from 2 to 100 for each month. The point at which the perplexity measure leveled off was used to indicate the optimal number of topics.

## Results

### Event Detection (Addressing R1)

**Figure 2:**
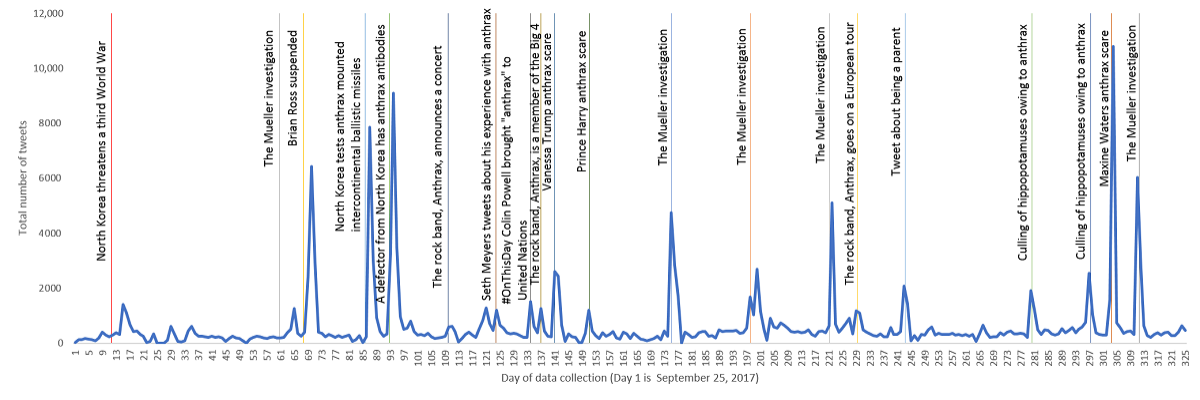
Line graph showing the number of tweets collected during each day of data collection (September 25, 2017, to August 15, 2018). Vertical lines indicate when news was first published about one of the detected anthrax-related discussions.

In total, 20 events were detected over the course of data collection (Figure 2), of which 6 concerned current anthrax-related events, 3 were about North Korea having access to anthrax, 3 were about anthrax scares, and 5 were related to the former director of the US Federal Bureau of Investigation (FBI) Robert Mueller, who oversaw the FBI during the anthrax attacks and was in charge of investigating the collusion with Russia during data collection. One was a news report about reporter Brian Ross being suspended owing to an erroneous report. Reporter Brian Ross was a topic of discussion because he also erroneously reported that Iraq and Saddam Hussein were responsible for the anthrax attacks, even though he was told that his story was inaccurate [26]. One event commemorated the anniversary of Colin Powell having brought a vial of “anthrax” to the United Nations, claiming that it is from Iraq. Culling of hippopotamuses owing to anthrax infection led to 2 events. Two were tweets that were highly retweeted: 1 from Seth Meyers about working at Saturday Night Live when they received the letter related to anthrax and another about using anthrax on the parents of bullies. Three events were announcements related to the band Anthrax, which were of no interest in this study and were removed by the relevance classifier.

The exact time of an event occurring could not be determined for all the detected events. Therefore, the time of report was based on when the original tweet was posted or when the weblink to the corresponding article or video in the tweet was posted (Table 1). Times between the original tweet and the start of the retweets was within minutes, as seen with the tweet from Seth Meyers, the tweet about being a parent, 2 tweets about the Mueller investigation, and a tweet announcing the band Anthrax is a member of the Big 4. Most tweets based on a new article started within a few hours of the article being posted. The longest time between an article and a tweet about the article concerned an article in The Federalist about former FBI Director Robert Mueller and a one about him “botching” investigations throughout his career.

**Table 1:** Time of the news report or the first tweet concerning a detected discussion, time of the first tweet discussing the news article or the first retweet, and the time between the event and its detection.

Event	Time of report	Time of detection	Time between report and detection
North Korea threatens a third World War	October 6, 2017, at 1:29 PM	October 6, 2017, at 5:35 PM	4 hours 6 minutes
The Mueller investigation	November 24, 2017, at 3 AM	November 25, 2017, at 5:02 PM	~1 day 15 hours
Brian Ross suspended	December 1, 2017 (clock time unknown)	December 1, 2017, at 4:14 PM	<24 hours
North Korea tests anthrax-mounted intercontinental ballistic missiles	December 19, 2017, at 7:32 PM	December 20, 2017, at 12:32 AM	5 hours
North Korean defector has anthrax antibodies	December 26, 2017, at 9:51 AM	December 26, 2017, at 2:53 PM	5 hours 2 minutes
Anthrax band announces a concert	January 11, 2018, at 3:47 AM	January 12, 2018, at 3:03 PM	~12 hours
Seth Meyers tweets about an anthrax experience	January 26, 2018, at 3:27 AM	January 26, 2018, at 3:28 AM	1 minute
#OnThisDay Collin Powell brought “anthrax” to the United Nations	February 5, 2018, at 9:46 AM	February 5, 2018, at 9:57 AM	11 minutes
Anthrax band is a member of the Big 4	February 8, 2018, at 2:40 AM	February 8, 2018, at 2:41 AM	1 minute
Vanessa Trump anthrax scare	February 12, 2018, at 10 AM	February 12, 2018, at 6:14 PM	~8 hours
Prince Harry anthrax scare	February 22, 2018, at 5:59 AM	February 22, 2018, at 10:58 AM	4 hours 59 minutes
The Mueller investigation	March 18, 2018, at 2:20 AM	March 18, 2018, at 2:20 AM	<1 minute
The Mueller investigation	February 8, 2018 (clock time unknown)	April 10, 2018, at 4:36 AM	~2 months
The Mueller investigation	May 3, 2018, at 9:59 PM	May 4, 2018, at 1:59 AM	4 hours
Anthrax band’s European tour	May 11, 2018, at 4 AM	May 11, 2018, at 8 AM	4 hours
Tweet about being a parent	May 25, 2018, at 11:34 AM	May 25, 2018, at 3:34 PM	4 hours
Culling of hippopotamuses owing to anthrax	July 1, 2018, at 9 AM	July 1, 2018, at 12:59 PM	3 hours 59 minutes
Culling of hippopotamuses owing to anthrax	July 18, 2018, at 3:09 AM	July 18, 2018, at 7:09 AM	4 hours
Maxine Waters anthrax scare	July 24, 2018, at 3:22 PM	July 24, 2018, 10:26 PM	7 hours 4 minutes
The Mueller investigation	August 1, 2018, 1:47 PM	August 1, 2018, at 1:47 PM	<1 minute

### Classification Performance (Addressing R2)

Initially, 204,008 tweets were collected. After preprocessing, 201,152 tweets remained. A random subset of 5000 unique tweets was manually labeled as “relevant” or “not-relevant” to *B. anthracis*. The distribution of relevant versus not-relevant tweets in the gold standard was uneven, with more relevant (n=3239 of 5000, 64.78%) than not-relevant tweets (n=1761 of 5000, 35.22%). The distribution of all relevant (n=165,844 of 201,152, 82.45%) vs not-relevant tweets (n=35,308 of 201,152, 17.55%) was also uneven, but with a larger proportion of relevant tweets. The difference in the ratio between the gold standard and final count is due to the gold standard including unique tweets, while several relevant tweets were retweeted numerous times in the actual data set.

The interrater reliability for relevancy was 0.87 (76% agreement) between raters. This indicates adequately high agreement [27]. Accordingly, a machine learning algorithm needed to be trained on the basis of the gold standard data set.

The performance metrics for the 4 machine learning algorithms on the gold standard are shown in Table 2. All algorithms had acceptable levels of performance (F_1_-score=0.72-0.80; precision=0.75-0.81; recall=0.75-0.81) with logistic regression analysis revealing an optimal performance with regard to precision (0.81), recall (0.81), and the F_1_-score (0.80).

**Table 2:** Precision, recall, and F_1_-score for the relevance machine learning algorithms with optimal performance on logistic regression analysis.

Machine learning algorithm	F_1_-score	Precision	Recall
Support vector machine	0.72	0.75	0.75
Random forest	0.78	0.78	0.79
Naïve Bayes classifier	0.79	0.79	0.79
Logistic regression	0.80	0.81	0.81

A confusion matrix was created by comparing the annotated data from the CBRNe expert to the sum of the predictions of each of the holdout sets (n=10). Most tweets were classified correctly through logistic regression analysis (true-positive=1116; true-negative=2931; false-positive=645; false-negative=308) compared to the support vector machine (true-positive=2993; true-negative=737; false-positive=1024; false-negative=246), random forest (true-positive=2802; true-negative=1120; false-positive=641; false-negative=437), and naïve Bayes classifier (true-positive=2566; true-negative=1361; false-positive=400; false-negative=637). The majority of misclassification was the algorithm classifying not-relevant tweets as relevant for logistic regression analysis (false-positive=645), the support vector machine (false-positive=1024), and random forest (false-positive=641), whereas the majority of misclassification was false-negative for the naïve Bayes classifier (false-negative=637).

An additional 500+1000 random, unique tweets not included in the gold standard were coded by the CBRNe expert and the logistic regression algorithm trained with the gold standard. Precision, recall, and the F_1_-score were determined between the expert’s and logistic regression’s codings and found to be adequately high (500: precision=0.65, recall=0.83, and F_1_-score=0.73; 1000: precision=0.58, recall=0.95, and F_1_-score=0.72). The substantial agreement indicates that the gold standard was a suitable representation of the entire corpus. The relevant tweets were further examined to determine how discussions on anthrax change over time and how anthrax-related events influence that discussion.

### Topical Analysis (Addressing R3)

#### Event-Related Topical Analysis

Of the 25 topics, 16 were related to the events detected (6 were about the Mueller investigation*,* 2 were about threats from North Korea, 3 were about an anthrax scare*,* and 2 were about culling of hippopotamuses, Seth Meyers, and being a parent) (Table 3). The topic of the Mueller investigation was discussed throughout 2018 and included tweets discussing perceived past failings of Former FBI Director Robert Mueller.

Threats from North Korea was a topic in September or October and December and concerned fear regarding North Korea threatening a third World War and reports a defector from North Korea who tested positive for anthrax antibodies.

During data collection, 3 anthrax scares were reported. The first 2 occur in February with regard to Prince Harry and 2 weeks later, with regard to Donald Trump Jr. The third scare occurred in July with regard to Representative Maxine Waters. These were called scares because all 3 letters or packages contained a powder, which was not anthrax.

The topic of culling of hippopotamuses includes tweets about culling of hippopotamuses in Namibia in September or October and Zambia in May owing to anthrax outbreaks in herds of hippopotamuses.

The last 2 events that were topics of discussion were Seth Meyers and being a parent. Seth Meyers was a highly retweeted tweet in February from Seth Meyers, which described his experience of working at Saturday Night Live when the anthrax attack occurred at the National Broadcasting Company. Being a parent was a tweet from a user who indicated why he/she was afraid to become a parent because he/she might transmit anthrax to the parents of bullies.

**Table 3:** Results of topic modeling for each month of data collection (September 25, 2017 to August 15, 2018) (N=26 topics).

Month	Topic
September and October	(#1) Threats from North Korea (#2) Responsible (#3) Culling of hippopotamuses
November	(#1) Vaccine (#2) Angela Merkel
December	(#1) Threats from North Korea (#2) India
January	(#1) Seth Meyers (#2) The Mueller investigation
February	(#1) New York Post (#2) Anthrax scare (#3) Anthrax scare (#4) Korean War
March	(#1) The Mueller investigation (#2) Travis Air Force Base
April	(#1) Abortion (#2) The Mueller investigation
May	(#1) The Mueller investigation (#2) Culling of hippopotamuses (#3) Being a parent
June	(#1) Cattle (#2) The Mueller investigation (#3) Abortion
July	(#1) Anthrax scare (#2) The Mueller investigation
August	(#1) The Mueller investigation

#### Non–Event-Related Topical Analysis

The remaining 10 topics were not detected events (2 about abortion, the New York Post, the Travis Air Force Base, India, cattle, Angela Merkel, responsible, vaccine, and the Korean War) (Table 3). “Abortion,” “New York Post,” and “Travis Air Force Base” concern scares. “India” and “cattle” both discuss natural anthrax outbreaks. “Angela Merkel” and “responsible” were both highly retweeted tweets. “Vaccine” and the “Korean War” both refer to controversies related to anthrax in the United States.

The 2 times “abortion” was a topic both discuss what it is like to work at an abortion clinic with the constant threats including an anthrax scare. “The New York Post” details a person’s experience working at the New York Post when they received a scare. “The Travis Air Force Base” discusses a suspicious package at the base and includes mentions of other events.

The topic “India” was the result of a research study in India, which reported that anthrax remains in the soil for 50-60 years. The topic “cattle” discusses a natural outbreak of anthrax in a herd of cattle in South Dakota.

“Angela Merkel” was a highly retweeted post that compared Angela Merkel to anthrax. Regarding “responsible,” a user jokingly asked how to tell someone they were responsible for anthrax attacks. 

“Vaccine” concerns the controversial anthrax vaccine. “Korean War” concerned the use of biological weapons by the United States during the Korean War. Both are controversial topics with “vaccine” being a topic of discussion throughout data collection.

## Discussion

### Event Detection (Addressing R1)

Of the 26 topics discovered over the 12 months of data collection, 12 were related to current anthrax events (3 about anthrax scares, 2 about threats from North Korea, and 7 about the Mueller investigation) (Multimedia Appendix 1). Seven topics were tweets that were highly retweeted (“responsible,” “Seth Meyers,” “New York Post,” “being a parent,” and “abortion”). Natural outbreaks were highlighted by 2 topics (“culling of hippopotamuses” and “cattle”). Two topics stemmed from responses to news articles (“Angela Merkel” and “India”). The topic “vaccine” stemmed from people who discussed the controversy regarding the armed forces requiring troops to be vaccinated against anthrax.

### Classification Performance (Addressing R2)

The majority of tweets concerned anthrax-related events. This class imbalance was in the random sample of labeled tweets and the total corpus, which shows that the gold standard was an accurate representation of the data. The relevance classifier performed well, with logistic regression analysis revealing an optimal performance. Error analysis revealed that the logistic regression classifier performs well with new data and was adequately generalizable to handle a large data set.

### Topical Analysis (Addressing R3)

#### Event-Related Topical Analysis

Although we were screening topics related to bioterrorism, natural outbreaks also trended as topics of discussion. The outbreaks discussed in this study resulted in a cull—selective killing of infected animals to prevent further disease spread—to prevent the spread of anthrax among hippopotamuses and cattle. While culls do not relate to terrorism, they can be controversial, which is why they emerged as topics [28]. While outrage and controversy were expressed in relation to both culls and attacks, tweets about culls are not useful for studying public reactions to bioterrorism-related anthrax events.

The topics “Seth Meyers,” “abortion,” and “New York Post” indicate that on anniversary dates or when similar events occur, people discussed the past use of weaponized anthrax or anthrax-related scares. One example was a discussion on the use of bioweapons by the United States during the Korean War. These topics also show that people tweet about their experiences with a past event when a similar event is occurring. All 3 tweets discussed how terrified they were and show that they are still affected, almost 2 decades later. Owing to discussions on past events during current ones, it will be important for government agencies to create a classifier to separate out tweets that discuss past events from those that discuss emerging events. However, these tweets will still need to be studied to inform how people might react to current events.

#### Non–Event-Related Topical Analysis

The topics “responsible,” “being a parent,” “Angela Merkel,” “vaccine,” and “India” show examples of what people discuss when an anthrax-related event is not occurring. When anthrax-related events are not occurring, people would still discuss new research findings, as demonstrated with the topic “India,” which included tweets that shared a news story wherein researchers found anthrax remains in the soil for 60 years. The emergence of “India” as a topic shows that people at risk of infection pay attention to news that might affect them.

Sometimes, joke tweets become viral, such as the one related to “responsible” joking about telling someone that they were responsible for the anthrax attacks, or another tweet about mailing anthrax to the parents of children who bullied their child. The people making these jokes do not understand the seriousness of anthrax or consider their risk to be nonexistent.

The topic “Angela Merkel” resulted from an article in a German newspaper where members of the Christian Democratic Union of Germany wanted Angela Merkel to resign because they disagree with her policies, and a person commented comparing Angela Merkel to anthrax. Tweets such as this one comparing someone to anthrax implies that the tweeter considers this person harmful, similar to calling someone “toxic.” These tweets likely do not indicate a threat but do indicate a large dislike or distrust of the person or group. Tweets wishing someone had anthrax or comparing someone to anthrax will remain topics for discussion when events are not occurring because of people expressing how much they dislike someone or how much they do not want to do something.

Tweets concerning the anthrax vaccine will be another topic of constant discussion owing to the controversy about its side effects. A majority (86%) of people who received the anthrax vaccine reported side effects, which led to some people to argue that the vaccine should be halted until one with fewer side effects is developed [29]. However, the Pentagon disagreed and stated that the current vaccine is the most reliable and safest way to protect service members. The dispute over the vaccine resulted in a constant stream of tweets throughout the year. Previous studies on vaccine sentiments can serve as a guide for the Pentagon to understand why people are so adversative to the vaccine, in order to address this fear [30-34].

### Usefulness of The Methods

Events were detected within 1-4 hours of the event, which was an improvement over previous studies [19-22]. While machine learning techniques were used specifically for detecting anthrax-related events, they are much more widely applicable; machine learning techniques could detect other terrorism-related events, answer other questions, or be used on other social media platforms. The usefulness of the methods is currently being demonstrated with the FBI’s search for the insurgents who invaded the Capitol building. The culprits could be identified by collecting and analyzing tweets containing the hashtags #stormthecapitol and #patriotparty and limiting tweets to those in English, which contain images or videos.

### Limitations

There are some limitations related to our data set and to using social media. These limitations include language constraints, the use of LDA, and bot accounts. These are standard limitations associated with infodemiology studies [35-39].

#### Demographics

The use of data from Twitter has an inherent sampling bias. Future studies could utilize other social media platforms to account for this bias. The search application programming interface only collects 1% of tweets from people with public profiles.

#### Language Constraint

Our data were restricted to tweets in English, which limits the generalizability of our findings. Limiting tweets to those in English limited the ability to study topics such as “culling of hippopotamuses” and “India,” the former having originated in Namibia and Zambia, and the latter in India. Future studies could address this limitation through the analysis of tweets in the prominent language spoken where the event occurred. Slang may have also affected our results. Anthrax is slang for smoking marijuana. Tweets using anthrax as slang were labeled as not-relevant, but some may have been misclassified. We may have also missed tweets discussing anthrax without using our keywords.

#### LDA

LDA has had some problems in the past with the number of revealed topics being greater than the number of true topics [40]. This was addressed by using perplexity and by combining topics covering the same tweet or topics that had ≥4 of the same most frequently used words. Before performing LDA, tweets were segregated into separate documents where each document included only tweets about that topic. All relevant tweets were separated by month before the LDA was performed on the tweets within that respective month.

#### Tweets By Bots

People who want to spread their message to as many people as possible program bots to spam their messages on social media platforms. If this is a concern for future studies based on these data, we would recommend checking each account to ensure that it is not a bot by using previously developed code or removing all duplicate and retweeted tweets to prevent bots from influencing such studies. In this study, tweets about the anthrax vaccine and Gulf War Syndrome, and about Matt Dehart, a hacker arrested by the FBI, who claimed that he was arrested and tortured to keep secrets, may have been from bot accounts [41]. Since this study aimed to provide a descriptive analysis of what people discuss about anthrax, and how these discussions relate to bioterrorism events, there was no need to attempt to identify bot accounts.

#### Interpretation of Peak Height

There is no guideline on what specific peak height indicates an event. A 3-fold spike was chosen because it allowed for the detection of all spikes related to anthrax events, without detecting spikes due to random noise. Future studies can start with this 3-fold spike but may need to adjust it on the basis of their results.

### Future Prospects

Future studies could further classify relevant tweets as discussing an event or not. This would help with misclassification being skewed towards false-positive findings and allow for a more detailed analysis of discussions about certain events.

To maximally harness this framework, future studies can utilize more social media platforms to eliminate the demographic bias, sample social media posts in all languages, which are related to a CBRNe event, and to identify and exclude bot accounts to determine what the general public thinks about an event. Other studies should focus on opinion tweets and exclude news stories, use these methods to analyze an actual anthrax attack, and study misconceptions or misinformation about anthrax. In this study, news posts were highly retweeted and skewed topics toward reports rather than people’s opinions. A study should also focus on social media platforms that people with extremist opinions use, to prevent incidents such as invasion of the Capitol.

### Conclusion

This was the first study to successfully create an automated tweet classification tool to analyze topics of discussion regarding anthrax related-events in real time. Through citizen sensing, detection time has decreased from 2 weeks to a few hours, advancing the field’s methodological capabilities for analyzing public discussions on CBRNe events. Our methods have been demonstrated to be effective and trustworthy for detecting discussions on anthrax-related events and classifying tweets as relevant or not-relevant. FBI analysts will be able to immediately detect CBRNe events using the framework of this study.

This study is important because it decreased detection time from a week to a few minutes to hours and developed a reliable and trustworthy framework that can be used for any CBRNe-related event. This will allow experts to address fear and misconceptions in real time, mitigating the additional damage that occurred after the anthrax attacks. Monitoring social media may also help rescuers locate people who may have left the scene before they could be decontaminated or properly treated. These methods could also help identify people involved in a terrorism incident if they take photographs or carry out a livestream, similar to what happened at the Capitol building.

## Appendix

Multimedia Appendix 1 Topics, keywords, and example tweets for each topic of discussion over the twelve months of data collection.

## Abbreviations

CBRNe: chemical, biological, radiological, and nuclear weapons or explosives
FBI: Federal Bureau of Investigation
LDA: latent Dirichlet allocation
