# Supplementary material for: Public Discussion of Anthrax on Twitter: Using Machine Learning to Identify Relevant Topics and Events
Source: JMIR Public Health Surveill. 2021 Jun 18;7(6):e27976. doi: 10.2196/27976 (PMC8277308; doi:10.2196/27976)
Supplement: Multimedia Appendix 1 [file publichealth_v7i6e27976_app1.docx]

Multimedia Appendix 1: Topics, keywords, and example tweets for each topic of discussion over the twelve months of data collection.

| Month | Topic | Words | Example Tweet |
| --- | --- | --- | --- |
| September and October | (#1) North Korean Threats | Smallpox, korea, north, weapon, war, mass, biological, produce, unleash, plague | North Korea threaten World War 3 with terrifying PLAGUE, ANTHRAX and SMALLPOX weapons... |
|  | (#2) Responsible | World, letter, someone, attack, body, responsible, noticeable, odor, one, report | RT: What's a good way to tell someone they have noticeable body odor, and that you were responsible for the anthrax letter attack… |
|  | (#3) Hippo Cull | Hippo, kill, suspect, outbreak, Namibia, dead, found, week, park, score | Suspected anthrax outbreak kills 107 hippos in Namibia's major game park in the Zambezi region in one week |
| November | (#1) Vaccine | US, military, troop, guinea, pigs, day, country, vaccine, regular, male | US Military Troops Used As Anthrax Guinea Pigs |
|  | (#2) Angela Merkel | Contact, ebola, shit, good, wrong, attack, case, ruin, Germany, fabric | Wrong???? They have ruined the very fabric of GERMANY.\n\nShe's anthrax... |
| December | (#1) North Korean Threats | Korea, north, antibody, defect, soldier, report, system, south, weapon | Report: North Korea soldier found to have anthrax antibodies - One of the North Korean soldiers who defected to the… |
|  | (#2) India | Threat, test, year, scare, soil, bacteria, area, agency, vaccine | No let-up in anthrax scare in Agency areas: Bacteria stays in the soil for 50-60 years - Times of India |
| January | (#1) Seth Meyers | Week, someone, sent, everyone, scare, month, nbc, third, snl, chill | RT @sethmeyers: This was my third week at SNL. It was a month after 9/11 and the week someone sent anthrax to NBC. Everyone was scared an… |
|  | (#2) The Mueller Investigation | Mueller, investigation, fbi, man, innocent, al, qaeda, prosecution, bush | RT: Mueller botched the Anthrax investigation and prosecution. \nHe should have been thrown on the trash heap of history then an… |
| February | (#1) New York Post | Sent, work, news, turn, outlet, ny, post, hoax, terrify, letter, fake | RT @maggieNYT: I worked at the NYPost when anthrax was sent to news outlets post 9/11 and it was terrifying. This turned out to be a hoax,… |
|  | (#2) Anthrax Scare | Powder, white, war meghan, markle, contain, threat, harry, biological, prince | Meghan Markle and Prince Harry in Anthrax threat terror |
|  | (#3) Anthrax Scare | Trump, jr, attack, mueller, Donald, one, threat, known, investigation | My son, Donald Trump Jr. , and his wife may have opened a letter containing anthrax. Some people are saying terrori… |
|  | (#4) Korean War | Post, korea, north, terrify, china, attack, wide, known, plague, left | U.S. use of biological warfare during the Korean War against Korea & China, included use of anthrax, plague and cho |
| March | (#1) The Mueller Investigation | Mueller, investigation, attack, botch, case, man, handle, open | RT: Mueller botched the Anthrax investigation and prosecution. \nHe should have been thrown on the trash heap of history then an… |
|  | (#2) Travis Air Force Base | Mail, attack, bomb, air, military, base, jr, scare, Austin, travis | RT: - Austin bombing\n\n- Don Jr. anthrax scare\n\n- Travis Air Force Base attack\n\n- Suspicious packages mailed to military installa… |
| April | (#1) Abortion | Work, year, investigation, attack, one, clinic, time, fake, letter, us | I worked in an abortion clinic during the time when Eric Rudolph was on the loose. After 9/11, our clinic received a fake anthrax letter. One of the doctors I worked with had an anti-choice activist follow his children home from school. He had Army of God propaganda on him. |
|  | (#2) The Mueller Investigation | Mueller, case, fbi, Robert, innocent, man, bulger, whitey, million, official | Mueller & Comey have history in FBI of lying to indict innocent people: Whitey Bulger case, anthrax, obviously Obam… |
| May | (#1) The Mueller Investigation | Mueller, back, case, men, people, qaeda, new, innocent, terror | RT: "When Comey and Mueller Bungled the Anthrax Case"- "Mueller, in Steven Hatfill the bureau had its man" AN INNOCENT MAN.… |
|  | (#2) Hippo Cull | Hippo, prevent, south, Zambia, company, deal announced, kill | #Zambia has announced that they are permitting 2,000 Hippos to be killed to prevent ‘anthrax’. \n\nHowever, this is just to conceal the fact that they have made a deal with a South African hunting company to allow more #TrophyHunting.\n\nCall on to REVERSE this! |
|  | (#3) Parent | Shit, self, control, uppercut, outta, parent, terrify, kid, mail | This is why I’m terrified to be a parent. I don’t know if I have enough self control to not uppercut the shit outta these kids & mail anthrax to their parents. |
| June | (#1) Cattle | South, Dakota, cattle, herd, confirm, us, animal, infect | Anthrax confirmed in South Dakota cattle herd |
|  | (#2) The Mueller Investigation | Mueller, investigation, attack, comey, botch, director, taxpayer, wrongful | 🚨Robert Mueller has been botching investigations since the anthrax attacks🚨\n\nA former FBI official involved in the investigation sued the FBI, alleging the FBI concealed exculpatory evidence.\n\nMueller held this case open for TEN YEARS! \n\n🚨MUST READ👇🏻 |
|  | (#3) Abortion | Scare, one, work, letter, fake, know, right, doctor | I worked for Planned Parenthood from 1999-2002.\n\nWe were firebombed once. We got an anthrax scare letter right after 9/11 (luckily fake). I was followed home once by a protester. We had multiple bomb threats. One doctor I worked with had protestors target his children at school. |
| July | (#1) Anthrax Scare | Maxine, waters, package, office, rep, label, left, mark, receive | BREAKING: Rep Maxine Waters' office has been evacuated after a package was received with the word "Anthrax" on it.\n\nReminder: Earlier this month Trump tweet-attacked her, calling her corrupt and 'low IQ"\n\nFACT: Trump is corrupt and Low IQ. |
|  | (#2) The Mueller Investigation | Mueller, attack, fake, case, send, package, walk, desperate | @FBI Comey and Mueller badly bungled the biggest case they ever handled. They botched the investigation of the 2001 anthrax letter attacks that took five lives and infected 17 other people, shut down the U.S. Capitol and Washington’s mail system, identified a "rogue scientist" who killed himself before his trial. |
| August | (#1) The Mueller Investigation | Mueller, uranium, many, fact, two, deal, corrupt, history, along, deal | I’m shocked that many are just now waking up to the fact Mueller oversaw two of the biggest sweetheart deals for sex offenders in our nation’s history, re Asimov & Epstein. Along w/the Uranium 1 deal, anthrax debacle that cost taxpayers $6 mil. He’s pure corruption personified. |
